# Clinicopathological Features and Prognosis of Metaplastic Breast Carcinoma: Experience of a Major Chinese Cancer Center

**DOI:** 10.1371/journal.pone.0131409

**Published:** 2015-06-26

**Authors:** Yiqian Zhang, Feng Lv, Yiling Yang, Xiaolong Qian, Ronggang Lang, Yu Fan, Fangfang Liu, Yaqing Li, Shuai Li, Beibei Shen, Gordon A. Pringle, Xinmin Zhang, Li Fu, Xiaojing Guo

**Affiliations:** 1 Department of Breast Pathology and Lab, Key Laboratory of Breast Cancer of Breast Cancer Prevention and Therapy, National Clinical Research Center of Cancer, Tianjin Medical University Cancer Institute and Hospital, Tianjin, China; 2 Department of Pathology and Laboratory Medicine, Temple University School of Medicine, Philadelphia, Pennsylvania, United States of America; INRS, CANADA

## Abstract

Metaplastic breast carcinoma (MBC) is a rare heterogeneous group of primary breast malignancies, with low hormone receptor expression and poor outcomes. To date, no prognostic markers for this tumor have been validated. The current study was undertaken to evaluate the clinicopathologic characteristics, the response to various therapeutic regimens and the prognosis of MBCs in a large cohort of patients from Tianjin Medical University Cancer Hospital in China. Ninety cases of MBCs diagnosed in our hospital between January 2000 and September 2014 were retrieved from the archives. In general, MBCs presented with larger size, a lower rate of lymph node metastasis, and demonstrated more frequent local recurrence/distant metastasis than 1,090 stage-matched cases of invasive carcinoma of no specific type (IDC-NST), independent of the status of estrogen receptor, progesterone receptor and human epidermal growth factor receptor 2 expressions. The five-year disease-free survival (DFS) of MBC was significantly worse than IDC-NST. Using univariate analysis, lymph node metastasis, advanced clinical stage at diagnosis, high tumor proliferation rate assessed by Ki-67 labeling, and epidermal growth factor receptor (EGFR) overexpression/gene amplification were associated significantly with reduced DFS, while decreased OS was associated significantly with lymph node metastasis and EGFR overexpression/gene amplification. With multivariate analysis, lymph node status was an independent predictor for DFS, and lymph node status and EGFR overexpression/gene amplification were independent predictors for OS. Histologic subtyping and molecular subgrouping of MBCs were not significant factors in prognosis. We also found that MBCs were insensitive to neoadjuvant chemotherapy, routine chemotherapy, and radiation therapy. This study indicates that MBC is an aggressive type of breast cancer with poor prognosis, and that identification and optimization of an effective comprehensive therapeutic regimen is needed.

## Introduction

Metaplastic breast carcinoma (MBC) is a rare type of breast cancer accounting for 0.2–5% of all invasive mammary carcinomas [1]. It encompasses a group of neoplasms characterized by differentiation of the neoplastic epithelium into squamous cells and/or mesenchymal-looking elements. The 2012 WHO Classification of Tumors of Breast divided MBC into subtypes based upon the morphologic components of the tumors. The majority of MBCs are triple negative for estrogen receptors (ER), progesterone receptors (PR) and human epidermal growth factor receptor 2 (HER2) expression (TNBC), and may express cytokeratin 5/6 (CK5/6) and/or epidermal growth factor receptor (EGFR). MBC tends to present as a large tumor mass with low axillary lymph node metastasis (LNM) and poor prognosis [2, 3]. The lack of ER, PR, and HER2 expression [2, 4, 5] makes endocrine therapy and molecularly targeted therapy ineffective, and therefore adjuvant and/or neoadjuvant chemotherapy have become the mainstay of management, although no therapeutic regimen has proven to be effective. So far, validated prognostic markers have not been identified for the tumor. Different morphologic and biologic features of the tumor have been reported in patients from different ethnic groups, and the outcomes of MBC diagnosed in different regions vary significantly [6]. Because of this variability and the fact that little is known about the biologic characteristics of MBC in the Chinese population, this study was undertaken to evaluate MBC with regard to its clinicopathologic characteristics, its response to multi-disciplinary therapeutic regimens and its prognosis in a large cohort of patients from Tianjin Medical University Cancer Hospital, a major Chinese cancer center.

## Materials and Methods

### Ethics statement

All human breast tissues were collected with written informed consent from patients prior to participation in the study. The protocols for collection and analysis of the samples were approved by the Institutional Review Board of the Tianjin Medical University Cancer Institute and Hospital, in accordance with the current revision of the Helsinki Declaration. The study was approved by the Institutional Review Board of the Tianjin Medical University Cancer Institute and Hospital.

### Case Selection

Ninety cases of MBC were identified from 30,216 cases of invasive breast carcinoma (0.3%) diagnosed between January 2000 and September 2014 at the Department of Breast Cancer Pathology and Research Laboratory, Tianjin Medical University Cancer Hospital, Tianjin, China. All patients were female and the median age was 54.6 years (range 28–89 years). Family history of breast cancer or BRCA gene mutation was noted in 4 of the patients. Prior to the cancer diagnosis, a portion of the patients had annual mammography or ultrasonography screening for breast cancer starting from age 40. Seventy-seven (85.6%) patients received modified or radical mastectomy (MRM), 5 patients (5.6%) accepted breast-conserving surgery, and the other 8 (0.9%) patients received quadrectomy. Final clean surgical margins in excisional specimens were achieved in all the patients, either initially or in follow-up wider excision or mastectomy.

The pathologic material for each case of MBC was retrieved from the archive of the department and reviewed. The diagnosis was verified independently in each case by 3 senior pathologists (L.F., R.L. and X.G.), using the criteria specified in the 2012 WHO Classification of Tumors of Breast [1]. In addition, 1,090 cases of invasive carcinoma of no special type (IDC-NST) were randomly selected from the same time period as the control group, among which 193 cases were triple negative for ER, PR and HER2 expression (TN-IDC).

### Immunohistochemistry

Immunohistochemistry (IHC) was performed on selected tumor sections using the avidin-biotin-immunoperoxidase technique for ER, PR, HER2, Ki-67, p53, CK5/6 and EGFR. All primary antibodies were purchased from Abcam (Cambridge, MA, USA). Formalin-fixed paraffin embedded tissue sections were employed in each case using a standard protocol. The immune-reaction was evaluated independently by the 3 pathologists.

The ER, PR and HER2 status was determined using the criteria of the American Society of Clinical Oncology/College of American Pathologists (ASCO/CAP) [7, 8]. For ER and PR, nuclear staining in ≥1% of the tumor cells was considered positive. HER2 immunoreactivity was evaluated on a standardized scale from 0–3 based on the intensity of membranous staining and the proportion of invasive tumor cells stained, with strong complete membranous staining in >10% of tumor cells (3+) considered positive. Ki-67 and p53 immunoreaction presented with nuclear staining, and CK5/6 and EGFR with membranous and/or cytoplasmic stain. Ki-67 labelling index was calculated and high tumor proliferation was defined as a labelling index ≥14% [9]. Overexpression of p53 was defined as nuclear stain in ≥10% tumor cells [10], and a cut-off of >10% tumor cells stained was adopted for CK5/6 positivity [11]. EGFR immunoreaction was evaluated based on the staining intensity, with 0 indicating absence of staining, and 1+, 2+, and 3+ representing respectively weak, moderate, and strong staining intensity. EGFR overexpression was defined as 2+ or 3+ staining. Molecular classification of tumors was performed using the established criteria [12]. Tumors negative for ER, PR and HER2, and positive for CK5/6 and/or EGFR were classified as basal-like carcinoma [13–15].

### Fluorescent in situ hybridization

Fluorescent in-situ hybridization (FISH) detection of HER2 gene amplification was performed in selected cases with equivocal IHC reaction for HER2 (2+), using FDA-approved PathVysion HER2 DNA Probe Kit (Abbott Laboratories). At least 20 invasive carcinoma cells in each case were evaluated to determine HER2 gene copies and the ratio of HER2 gene vs chromosome 17 centromere signals. HER2/CEP17 ratio >2.0 was considered positive HER2 gene amplification, per the 2013 ASCO/CAP recommendations [8]. FISH for EGFR was performed using the LSI EGFR/CEP 7 Probe (Vysis) per manufacturer's instruction. EGFR gene amplification was defined as a ratio of EGFR gene vs chromosome 7 centromere signals ≥2.0. EGFR FISH-amplified samples also included those with ≥40% of tumor cells demonstrating ≥4 copies of EGFR gene.

### Survival analysis

All patients were followed up for 1–173 months with a median of 59 months. They were followed at 3-month intervals initially, then at 6-month intervals, and annually afterwards. Two patients were lost in follow-up. Patients were censored from the date of last follow-up visit or death from causes other than breast cancer, local or regional recurrences, or the development of a second primary carcinoma, including contralateral breast cancer. If a patient was confirmed to have metastasis during follow-up without recurrence, the last follow-up visit date was used. Age, time to first recurrence, and survival time were calculated relative to the primary diagnosis date. Kaplan–Meier survival curves were constructed, and between-group differences were tested using the log-rank test. The relative importance of potential prognostic variables was tested using Cox-proportional hazard analysis and expressed with a 95% confidence interval (CI).

### Statistical analysis

The statistical analysis was performed with the use of software packages SPSS version 19.0. All statistical tests were two-sided, and a P value of <0.05 was considered significant.

## Results

### Clinicopathological features

Characteristics of the 90 MBC patients and control patients are summarized in Table 1. Compared to patients with IDC-NST or TN-IDC, patients with MBC displayed unique characteristics, such as larger tumor size (p = 0.000; p = 0.013) and less frequent lymph node metastasis (LNM; p = 0.000; p = 0.000). No significant differences in patient age and tumor stage were identified. As reported previously, a greater proportion of MBCs are TNBCs than tumors in the IDC-NST group (p = 0.000).

**Table 1:** Clinicopathological features of MBC, IDC-NST and TN-IDC.

Characteristics		MBC	IDC-NST	P	TN-IDC	P
		N(%)	N(%)		N(%)	
**Age**	≤50	35(38.9)	502(46.1)	0.189	96(49.7)	0.088
	>50	55(61.1)	588(53.9)		97(50.3)	
**LN metastasis** *	Yes	16(20.8)	541(50.8)	0.000	92(49.2)	0.000
	No	61(79.2)	523(49.2)		95(50.8)	
**Primary tumor**	1	19(21.1)	348(31.9)	0.000	47(24.4)	0.013
	2	51(56.7)	674(61.8)		128(66.3)	
	3	19(21.1)	50(4.6)		14(7.3)	
	4	1(1.1)	18(1.7)		4(2.1)	
**Recurrence/metastasis**	Yes	22(24.4)	136(12.5)	0.001	29(15.0)	0.044
	No	66(73.3)	954(87.5)		164(85.0)	
**Pathologic tumor stage**	I	12(13.3)	221(20.3)	0.061	32(16.6)	0.062
	II	68(75.6)	688(63.1)		120(62.2)	
	III	10(11.1)	181(16.6)		41(21.2)	
**Type of surgery**	MRM	77(85.6)	1064(97.6)	0.000	187(96.9)	0.000
	Other	13(14.4)	26(2.4)		6(3.1)	
**Chemotherapy**	Yes	74(82.2)	870(79.8)	0.583	172(89.1)	0.109
	No	16(17.8)	220(20.2)		21(10.9)	
**Radiotherapy**	Yes	5(5.6)	222(20.4)	0.001	52(26.9)	0.000
	No	85(94.4)	868(79.6)		141(73.1)	
**Endocrine therapy**	Yes	9(10.0)	512(47.0)	0.000	_	_
	No	81(90.0)	578(53.0)		_	
**Anti-HER2 therapy**	Yes	1(1.1)	127(11.7)	0.000	_	_
	No	89(98.9)	963(88.3)		_	
**TNBC**	Yes	64(71.1)	193(17.7)	0.000	_	_
	No	26(28.9)	897(82.3)		_	

MBC, Metaplastic breast carcinoma; IDC-NST, Invasive carcinoma of no special type; TN-IDC, Triple negative invasive ductal carcinoma; MRM, Modified radical mastectomy; TNBC, Triple negative breast cancer;

* No lymph node information was available in 13 of the MBC cases in file.

Malignancy was diagnosed in 59 of 68 (86.8%) patients with MBC in preoperative ultrasonographic evaluation. Calcifications were found in 48 patients (48/69, 69.6%) in mammographic evaluation. Histologic diagnosis of metaplastic carcinoma was only achieved in 4 of 34 patients (11.8%) in preoperative core needle biopsies (Table 2).

**Table 2:** Preoperative evaluation of MBC patients.

Characteristics		Number	Percentage %
**Ultrasonography diagnosis**	Malignant	59	86.8
	Benign	4	5.9
	Other results	5	7.3
**Mammography**	Calcification	48	69.6
	No calcification	21	30.4
**Core needle biopsy diagnosis**	Invasive carcinoma	16	47.1
	Metaplastic carcinoma	4	11.8
	Other results	14	41.1

Seventy-seven (85.6%) patients received modified or radical mastectomy (MRM) and 5 patients (5.6%) accepted breast-conserving surgery. Three patients (1 spindle cell carcinoma, 1 squamous cell carcinoma, and 1 carcinoma with cartilaginous metaplasia) received neoadjuvant chemotherapy, but all failed with no reduction in tumor size observed. Postoperatively, paclitaxel plus anthracycline adjuvant chemotherapy was offered to 74 of the patients (82.2%), 3 patients (3.3%) received one of the neoadjuvant chemotherapeutic regimens (Paclitaxel+ epirubicin, or epirubicin + cyclophosphamide + docetaxel), and 5 patients (5.6%) had radiotherapy. Nine patients (10%) with ER and/or PR positive tumors accepted endocrine therapy, and 1 patient with tumor HER2 overexpression (1.1%) received adjuvant trastuzumab. Fewer patients in the MBC group received MRM (p = 0.000; p = 0.000), radiotherapy (p = 0.000; p = 0.001), endocrine therapy (p = 0.000) and anti-HER2 therapy (p = 0.000) than those in the TN-IDC and/or IDC-NST group (Table 1).

### Histopathological features

The average tumor size of MBC was 4.01 cm (1.0–10.0 cm). LNM was identified in 16 of 77 cases (20.8%) with regional lymph node biopsy. The most common MBC subtype was spindle cell carcinoma (34.4%), followed by squamous cell carcinoma (31.1%) and MBC with mesenchymal differentiation (24.5%). Fibromatosis-like subtype (4.4%) was the least common in this cohort of patients (Table 3). Among them, squamous cell carcinoma had the highest lymph node metastasis rate (25.9%, data not shown).

**Table 3:** Histologic subtypes of MBC.

Histologic subtypes	Number	Percentage %
**Spindle cell carcinoma**	31	34.4
**Squamous cell carcinoma**	28	31.1
**Metaplastic carcinoma with mesenchymal differentiation**	22	24.5
**Mixed metaplastic carcinoma**	5	5.6
**Fibromatosis-like metaplastic carcinoma**	4	4.4

### Molecular Classification

Two cases were classified as luminal A type (2.2%), 17 cases were luminal B type (18.9%), 7 cases were HER2-overexpression type (7.8%), and 64 cases were triple-negative type (71.1%). Fifty eight cases (85%) of the triple negative tumors were basal-like type breast carcinomas (Table 4).

**Table 4:** Molecular classification of MBC based upon the major component.

Molecular Classification	Number	Percentage %
**Luminal A**	2	2.2
**Luminal B**	17	18.9
**HER2-overexpression**	7	7.8
**Triple-negative**	64	71.1
**(Basal-like)**	58	64.4

### EGFR overexpression and copy number analysis

By immunohistochemistry, EGFR overexpression was identified in 52 of the 90 (57.8%) cases, and in 46 of the 64 triple-negative (71.9%) carcinomas. Squamous cell carcinomas had a significantly higher proportion of EGFR overexpression (82.1%), compared to other subtypes (p = 0.002; Table 5).

**Table 5:** EGFR overexpression of MBC.

Subtype of MBC	EGFR		P
	-/1+	2+/3+	
**Squamous cell carcinoma**	5	23	
**others**	33	29	0.002

**Fig 1:**
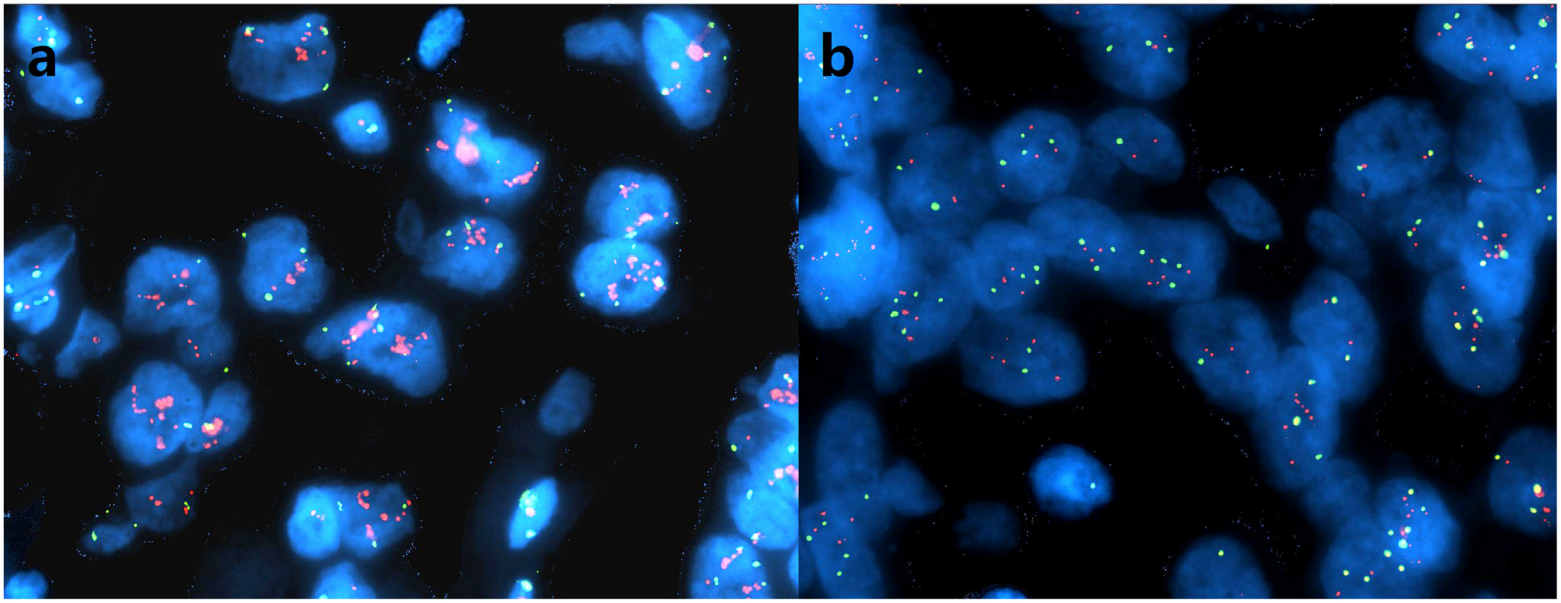
FISH analysis on EGFR gene amplification in MBC. Representative images of amplification of EGFR (a) and high aneusomy of EGFR (b). EGFR gene amplification was defined as a ratio of EGFR gene vs chromosome 7 centromere signals ≥2.0. EGFR FISH-amplified samples also included those with ≥40% of tumor cells demonstrating ≥4 copies of EGFR gene.

Forty-seven of 52 cases with EGFR overexpression were submitted for FISH analysis, and 14 of them (29.8%) demonstrated EGFR gene amplification (Fig 1a and 1b). Of these 14 cases, 5 (35.7%) showed increased EGFR copy number and 9 (64.3%) displayed high aneusomy.

### Survival analysis

Recurrence and/or distant metastasis occurred in 22 patients (24.4%) during follow-up. The most common site of recurrence was the chest wall and the most common metastatic site was lung, followed by bone and brain. A 67.9% five-year DFS and a 78.7% of five-year OS were identified in this cohort of patients. In univariate analysis (Table 6), LNM (p = 0.000), advanced stage at diagnosis (p = 0.047), EGFR overexpression (p = 0.007), and high Ki-67 labeling (p = 0.026) of tumors were the significant predictive factors for reduced DFS, among which only LNM (p = 0.008) was significant in multivariate analysis. For OS, tumor lymph node metastasis (p = 0.000), EGFR overexpression (p = 0.049), and gene amplification (p = 0.002) were the significant predictive factors in univariate analysis, among which lymph node metastasis and EGFR gene amplification were significant predictive factors using multivariate analysis (p = 0.001 and 0.022). Tumor size, morphologic subtype, biologic marker (ER, PR and HER2) expression, and therapeutic regimen were not significantly associated with patient survival.

**Table 6:** Univariate analysis and multivariate analysis of MBC patient’s survivals.

*Characteristics*		*DFS*				*OS*			
		Univariate		Multivariable		Univariate		Multivariable	
		5-yearDFS %	P	HR (95% CI)	P	5-yearOS %	P	HR (95% CI)	P
**Age**	≤50	72.9	0.280			86.1	0.272		
	>50	64.4				73.2			
**T stage**	≤5cm	70.4	0.805			82.4	0.549		
	>5cm	62.6				71.7			
**N stage**	Negative	77.4	0.000	1.473–12.846	0.008	88.8.	0.000	2.175–24.631	0.001
	Positive	29.2				44.2			
**Stage**	I	73.2	0.047	0.198–1.550	0.261	87.5	0.065		
	II	62.3				78.1			
	III	41.7				60.0			
**Feature of MBC**	Spindle	71.8	0.982			76.2	0.804		
	Squamous	63.4				75.5			
	Mesenchymal	69.2				80.8			
	Fibromatosis-like	66.7				100			
	Mixed	66.7				100			
**ER**	Negative	64.5	0.237			77.7	0.783		
	Positive	82.5				82.5			
**PR**	Negative	67.7	0.945			80.8	0.348		
	Positive	68.2				68.2			
**HER2**	Negative	68.2	0.886			76.6	0.984		
	Positive	66.7				88.9			
**Ki67**	0(0%-14%)	100	0.026	0.596–5.009	0.314	100	0.131		
	1(14%-50%)	83.4				87.1			
	2 (≥51%)	51.1				69.1			
**P53**	<10%	80.8	0.179			85.4	0.324		
	≥10%	58.1				73.6			
**CK5/6**	≤10%	63.8	0.673			78.7	0.859		
	>10%	70.1				78.5			
**EGFR**	- /1+	87.1	0.007	0.920–9.084	0.069	95.0	0.049	0.596–12.866	0.194
	2+/3+	54.7				68.4			
**EGFR gene**	No amplification	73.5	0.103			84.5	0.002	1.209–11.904	0.022
	Amplification	44.0				54.5			
**Chemotherapy**	Yes	64.5	0.445			76.1	0.237		
	No	83.6				90.0			
**Endocrine**	Yes	76.2	0.722			87.5	0.654		
	No	66.4				77.4			
**Operation**	MRM	66.8	0.711			77.7	0.656		
	Other	80.8				87.5			

DFS, disease-free survival; OS, overall survival; ER, estrogen receptors; PR, progesterone receptors; HER2, human epidermal growth factor receptor 2; CK5/6, cytokeratin 5/6; MRM, modified radical mastectomy

Although not statistically significant, prognostic trends for certain morphologic subtypes of MBC were noted. Four patients with fibromatosis-like MBC had an average DFS of 64 months and OS of 82 months, in contrast to a DFS of 51 months and OS of 55 months for patients with other histopathologic subtypes. Two patients with luminal A type MBC showing mesenchymal differentiation had an average DFS of 76 months and OS of 76 months, in contrast to a DFS of 51 months and OS of 56 months for patients with other molecular subtypes.

During the same period of follow-up, development of local recurrence or distant metastasis was identified in 136 patients (12.5%) with IDC-NST and in 29 patients (15.0%) in the TN-IDC group (Table 1), significantly less than those with MBC (24.4%) (P = 0.001, P = 0.044, respectively).

**Fig 2:**
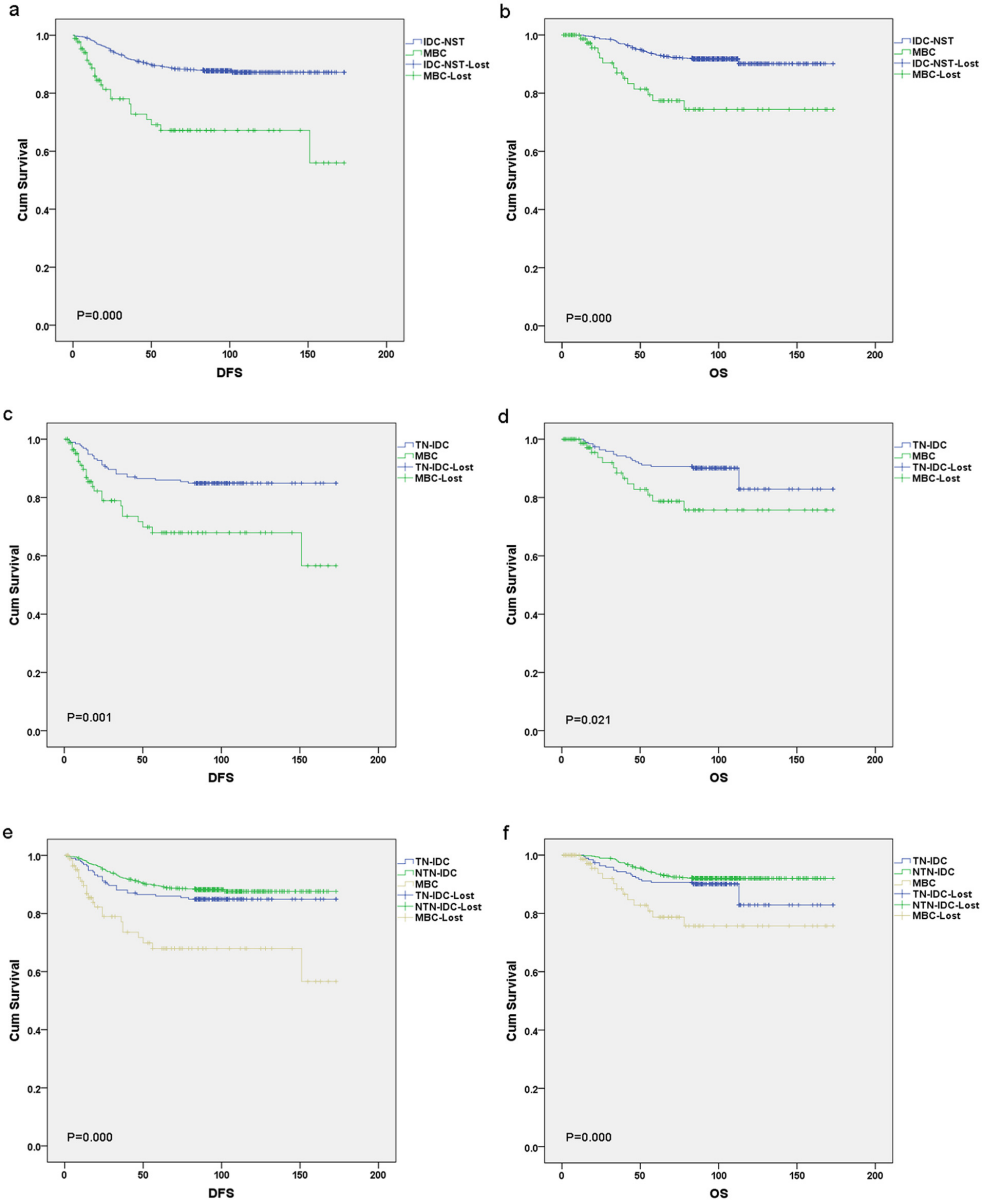
Patient’s survival curves of MBC, IDC-NST and TN-IDC. Patients with MBC demonstrated shortened five-year DFS and five-year OS, when separately compared with those in the IDC-NST (a and b) and TN-IDC groups of patients (c and d). Group comparison analysis among MBC, TN-IDC and non-triple negative IDC (NTN-IDC) showed that patients with MBC had the worst five-year DFS and OS followed by TN-IDC, while NTN-IDC carried the most favorable five-year DFS and OS among the groups (e and f).

Patients with MBC demonstrated a shorter five-year DFS (67.9% vs 88.9% vs 86%; p = 0.000, p = 0.001 respectively), and five-year OS (78.7% vs 93.0% vs 90.6%; p = 0.000, p = 0.021 respectively), when compared separately with survival in the IDC-NST and TN-IDC groups of patients (Fig 2a–2d). Group comparison analysis among MBC, TN-IDC and non-triple negative IDC (NTN-IDC) showed that patients with MBC had the worst five-year DFS and OS, followed by TN-IDC and NTN-IDC, which carried the most favorable five-year DFS (p = 0.000) and OS (p = 0.000) among the groups (Fig 2e and 2f).

## Discussion

Previous studies have suggested that patients with MBC tend to present with larger tumors, less lymph node involvement, a higher proportion of triple-negative cases, and an increased rate of distant metastasis, compared to IDC-NST [2, 3]. MBC has been reported to have a poorer prognosis than IDC [3, 16] although comparable prognoses have been reported in cases with matched stages. In this cohort of patients, with no significant difference in tumor stage among the groups of MBC, IDC-NST and TN-IDC. MBC had the largest average tumor size, the lowest rate of LNM, and the highest frequency of local recurrence and/or metastasis, and the worst prognosis measured by five-year DFS and OS. The results indicate that MBC has a poorer prognosis than that of IDC-NST and TNBC.

The 3-year DFS of MBC patients varies from 15% to 76% and the 3-year OS from 48% to 91% [5, 17–19]. With longer follow-up, we identified a 67.9% five-year DFS and a 78.7% five-year OS in our patients. Our findings are similar to those reported by Base et al in Korean patients [4] and Gultekin et al in a Turkish population [17]. Multiple factors may contribute to the wide range of survival, including differences in patient population, classification of tumors, stage of tumors at presentation, patient management, and data collection.

The 90 cases of MBC culled from over 30,000 cases of invasive breast carcinomas (0.3%) diagnosed in the past 15 years in this cancer center of China showed that average tumor size and percentage of TNBC were similar to those reported in the literature [2, 3, 5, 17, 20]. Noteworthy was the finding that the rates of lymph node metastasis (20.8%) and recurrence/distant metastasis (24.4%) were lower in our patient population than the rates of lymph node metastasis (24%-35%) [3, 5, 17] and recurrence/metastasis (26.9%-60%) reported in the literature [2, 16, 21].

MBC is a heterogeneous group of malignant breast tumors consisting of different morphologic subtypes with the frequency of subtypes showing considerable variation in different patient populations. Rakha et al [6] found that the most common subtype of MBC in Western countries was spindle cell carcinoma (34%), while squamous cell carcinoma (34%) was the most common in patients from Hong Kong and Singapore. Lai et al [3] reported that squamous cell carcinoma (35.6%) was the most common subtype in Taiwan, followed by carcinomas with osseous/chondroid differentiation (24.4%), sarcomatoid carcinoma (20%) and spindle cell carcinoma (8.9%). Luini et al [20] showed that MBC with matrix-production was the most common subtype (45.9%) in European patients, followed by carcinosarcoma (24.3%) and squamous cell carcinomas (18.9%). Using the current WHO classification, we found that spindle cell carcinoma (34.4%) is the most common subtype of MBC in the Chinese population, followed by squamous cell carcinoma (31.1%) and carcinoma with mesenchymal differentiation (24.5%), similar to that of Western patients [6]. While the variation in frequency of subtypes in different patient populations around the world is intriguing, variation in tumor classification and small case numbers in each study may be the principal factors contributing to the inconsistent frequency of tumor subtypes in the different studies. Further data collection is still needed to build up a valuable universal database.

Accurate diagnosis of MBC can be a challenge in preoperative core needle biopsies [21]. In our study, accurate diagnosis was made in only 11.8% of the cases. Tumor heterogeneity is presumably the major contributing factor to the challenge. Therefore, surgical excision is the necessary procedure to achieve the final diagnosis, and the choices include mastectomy (modified or radical mastectomy MRM), lumpectomy, and breast-conserving surgery (BCS) [22]. Because MBC patients typically present with a large mass, MRM was often performed [3, 23, 24]. The selection of surgical procedure may impact the 5 years-DFS of patients [16].

So far, there are no validated prognostic markers for MBC. Lee et al [16] reported that the subtype of MBC (non-squamous cell carcinoma vs squamous cell carcinoma) was associated with DFS, but its association with OS was not identified. Lester et al [25] found sarcomatoid MBCs were more aggressive than other triple-negative cancers. Sanguinetti et al [26] found tumor size had an important impact on patient outcome. Rakha et al [6], in their recent study of 405 MBC patients from a large international multicenter series, found that lymph node stage, lymphovascular invasion, histologic subtype, and chemotherapy were associated with lower breast cancer-specific survival and/or disease free interval. They found that spindle cell carcinoma had particularly aggressive biological behavior. In addition, significant differences in clinicopathologic features of MBC were described in patients from Western countries and from Asian countries, and they observed that MBC in Asian countries had a more favorable prognosis. Several of our findings differ from those of Rakha. In our cohort, the spindle cell carcinoma was found to be the most common subtype of MBC and it did not lead to a worse DFS or OS. Also, we couldn’t find a significant association between the histologic subtype of MBC and patient prognosis. However, we did find that tumor LNM, stage, proliferation rate measured by Ki-67 staining, and EGFR overexpression were significantly associated with patient DFS in a univariate analysis. Among these factors, the status of lymph node metastasis was an independent prognostic factor for DFS in multivariate analysis. We also found that the rates of lymph node metastasis and EGFR gene amplification were significantly associated with patient OS both in univariate and multivariate analyses. However, tumor size, the status of tumor biomarkers (molecular subtypes), operative procedure, and postoperative therapy were not significant prognostic factors.

The inability of standard therapeutic protocols to effectively treat MBC has prompted a search for other therapeutic options, including those targeting EGFR. Aberrant signaling through EGFR overexpression is associated with neoplastic cell proliferation, migration, stromal invasion, resistance to apoptosis, and angiogenesis [27]. Bae et al [4] reported that MBC exhibited higher expression of EGFR compared to triple negative infiltrating ductal carcinoma. Reis-Filho et al [28] observed that 19 of 25 (76%) MBC cases exhibited EGFR expression. In our study, EGFR overexpression was identified in 57.8% of MBCs, and in 71.9% of the triple negative MBCs. Basal-like MBC lacking EGFR and KIT activating mutations may exhibit high EGFR copy numbers [29]. Reis-Filho al [28] reported EGFR gene amplification in 37% of the MBCs with EGFR overexpression. We found that 29.8% (14/47) of MBCs demonstrated EGFR gene amplification. These results beg the question of whether MBC patients with EGFR overexpression and/or gene amplification might benefit from EGFR tyrosine kinase inhibitor and EGFR monoclonal antibody (cetuximab) therapies. We noted that squamous cell carcinoma had a significantly higher proportion of EGFR overexpression (82.1%) compared to other subtypes (p = 0.002). Whether squamous cell carcinoma will respond more favorably to cetuximab remains a valid clinical question to be answered. It seems reasonable to recommend a routine assessment of the EGFR status in MBC and to further explore this therapeutic option.

In summary, we report the clinicopathologic features and prognostic predictive markers in a large cohort of MBC patients from a major Chinese cancer center, and some unique features of MBC in the Chinese population have been noted. LNM is identified as an independent predictive factor for unfavorable DFS, and LNM and EGFR overexpression/gene amplification are independent predictive factors for decreased OS. This study indicates that MBC is an aggressive type of breast cancer with poorer prognosis than IDC-NST and TN-IDC. New therapy targeting EGFR in tumors with overexpression and/or gene amplification of EGFR is worthy of further exploration.
